# Cognitive safety under epicranial cortex stimulation of the epileptic focus

**DOI:** 10.1002/epi4.70216

**Published:** 2026-01-31

**Authors:** Kathrin Wagner, Iris Gorny, Julia Kustermann, Jessika Paula Losacker, Birgitta Metternich, Julia Berg, Bastian E. A. Sajonz, Volker Arnd Coenen, Elisabeth Kaufmann, Susanne Knake, Andreas Schulze‐Bonhage

**Affiliations:** ^1^ Department of Neurosurgery, Epilepsy Center Medical Center, University of Freiburg Freiburg Germany; ^2^ Department of Neurology, Faculty of Medicine, Epilepsy Center Hessen Philipps‐University Marburg Marburg Germany; ^3^ Department of Neurology, Epilepsy Center Muenchen University Hospital, Ludwig Maximilian University of Munich Munich Germany; ^4^ Department of Stereotactic and Functional Neurosurgery Medical Center, University of Freiburg Freiburg Germany; ^5^ LOEWE Research Cluster for Advanced Medical Physics in Imaging and Therapy (ADMIT) TH Mittelhessen University of Applied Sciences Giessen Germany

**Keywords:** eloquent cortex, executive function, memory, neurostimulation, noninvasive

## Abstract

Epicranial focal cortex stimulation (FCS) is a new CE‐certified treatment for pharmacoresistant focal epilepsy. In a multicenter observational trial, we report cognitive tolerability in 11 patients with pharmacoresistant epilepsy undergoing epicranial focal cortex stimulation (eFCS) over the predominant seizure focus (left temporal (*N* = 4), central (*N* = 2 right, *N* = 1 left), left frontal (*N* = 2), left temporoparietal (*N* = 1), and left frontoparietal (*N* = 1)). Extensive cognitive assessment focused on domains represented in the stimulated areas and was compared between baseline and first and last follow‐up (after a median 35 months of stimulation). Based on the reliable change index, the majority of patients remained cognitively stable; 3/11 patients showed improvements in more than one cognitive parameter at both follow‐up assessments. Isolated task parameters deteriorated exclusively at one time point; no patient revealed consistent worsening. Cognitive changes did not correspond to stimulation site or seizure outcome. Overall, the results from this case study provide preliminary evidence of stable cognitive functioning under focal cortical stimulation even if performed over eloquent brain regions. These findings are limited by the patient sample size and medication changes in part of the patients and need corroboration in larger patient cohorts.

**Plain Language Summary:**

We report on preliminary results of a new brain stimulation treatment for epilepsy that doesn't respond to medication. Over about 3 years, 11 patients showed stable cognitive performance, some even improved, and no patient had lasting declines. These results provide preliminary evidence that the treatment is generally safe for brain function. However, larger studies are needed to confirm these findings.

**Key points**

- Epicranial FCS was applied to 11 patients with pharmacoresistant focal epilepsy over eloquent brain regions.
- Cognitive assessments over ~35 months showed most patients remained cognitively stable.
- Cognitive changes were independent of stimulation site or seizure outcome; findings need larger studies.

## 1 INTRODUCTION

Not all patients with pharmacoresistant focal epilepsy are eligible candidates for resective surgery of the epileptogenic brain region. One reason can be elevated surgical risks due to localization or eloquence of the epileptogenic brain region. The newly approved method of epicranial focal cortex stimulation (eFCS) might be a promising alternative to achieve reduction in seizure frequency in patients with drug‐refractory focal epilepsy.^1^, ^2^ Very little is known about the cognitive safety of this treatment. Winter et al.^3^ presented preliminary results of improvements in visuoconstruction, verbal and figural fluency, and short‐term memory during 12 months of neurostimulation in 15 patients.

The aim of the present study is to investigate the cognitive performance before and after implantation of epicranial electrodes and after several months of stimulation in a multicenter observational trial.

## 2 METHODS

### 2.1 Patients

Eligible patients were aged 18 to 75 years, with focal‐onset seizures uncontrolled by at least two antiseizure medications (ASMs). Patients had three or more seizures per month and a predominant epileptic focus that was temporolateral or extratemporal in origin. Within the clinical trials,^1^ 14 patients (9 from Freiburg, 4 from Marburg, 1 from Munich) were implanted with epicranial electrodes for eFCS with the EASEE® system and received extensive neuropsychological assessments between 02/2019 and 12/2020. For a detailed description of the procedure see.^1^ The study was approved by the local ethics committees and each patient gave informed written consent. Three patients had to be excluded from the analysis (due to skull metal implant, technical problems, and suspicion of an additional neurodegenerative disease). Subsequently, we report on 11 patients who underwent detailed neuropsychological examinations before and on average 7.4 months (SD = 0.5; Median = 7 months), and 26.1 months (SD = 11.1; Median = 35 months) after implantation.

### 2.2 Neuropsychological assessment

In all patients, the EpiTrack® was administered,^4^ which is a short and repeatable neuropsychological screening test with a parallel version to assess attention, executive functions, and working memory with six different tasks: psychomotor speed, mental flexibility, response inhibition, verbal fluency, problem solving/anticipation, and working memory. In addition, further test procedures were applied that were adapted to the stimulation site (i.e., special focus on language and memory functions with left‐sided temporal stimulation and additional examination of fine motor skills with stimulation of the central region, for an overview see Table 1).

**TABLE 1:** Tests applied according to stimulation site.

Stimulation site	Cognitive domain		Applied test (parameter)
All		Attention, executive functions, and working memory	EpiTrack® Score
	Attention	Visuomotor speed	Trail‐Making‐Test part A (time in s)
	Executive functions	Cognitive flexibility	Trail‐Making‐Test part B (time in s)
	Memory	Auditory working memory	Subtest working memory EpiTrack® (max. digit span backwards)
		Verbal learning and delayed free recall	Verbaler Lern‐ und Merkfaehigkeitstest (German version of the Rey Auditory Verbal Learning Test, sum of correctly recalled words in five learning trials and correctly recalled words after 20 min)
	Language	Lexical verbal fluency	Subtest lexical verbal fluency EpiTrack® (sum of correctly produced words)
Temporal	Language	Naming	Boston Naming Test (sum of correctly named pictures)
Frontal	Language	Semantic verbal fluency	Regensburger Wortfluessigkeitstest (semantic controlled oral word association, sum of correctly produced words)
	Executive functions	Figural fluency	5‐Points‐Test, flexibility score (percentage perseverative errors of total designs)
		Interference	Subtest interference EpiTrack® (time in s)
		Visual planning	Subtest maze interference EpiTrack® (time in s)
Central	Motor functions	Fine motor speed	Purdue Pegboard: unimanual, bimanual, and assembly (sum of items)
Parietal	Visuspatial function	Visual working memory	Wechsler Memory Scale block spans (max. block span backwards)
		Visuoconstruction	Block Design (Wechsler Adult Intelligence Scale‐IV, raw score)

Reliable change indices (RCI) were calculated for each of the test parameters as follows^5^:

$$\mathrm{RCI}=\left(X_{2}-X_{1}\right)/{\mathrm{SE}}_{\text{diff}}$$

where *X*_1_ is the baseline score, *X*_2_ is the follow‐up score, and SE_diff_ (standard error of the difference) is: SE_diff_ = √(2·(SEM)^2^) = SD√(2(1−*r*)).

SEM is the standard error of measurement (SEM = SD√(1 − *r*)), SD is the baseline standard deviation, and r is the test's reliability coefficient (e.g., Cronbach's alpha). A reliable change (RCI ≥1.96 or ≤ −1.96 at 95% confidence) indicates the score shift exceeds measurement error. Positive/negative values denote improvement/deterioration. Cognitive outcome at follow‐up under stimulation was then classified as unchanged, improved, or deteriorated as compared to baseline assessment.

Furthermore, test variables assessed for all patients were analyzed using non‐parametric statistic Friedman‐tests due to the small sample size to look for significant overall changes across the three time points (effect sizes were calculated using Kendall's W). The analyses included the variables EpiTrack® Score, TMT‐A and ‐B, digit span backwards, verbal learning, verbal delayed recall, and lexical fluency. When the Friedman‐test indicated a significant overall effect, post‐hoc pairwise comparisons were performed using Wilcoxon signed‐rank tests with Holm correction to adjust for multiple comparisons.

### 2.3 Trial design

Patients were implanted and the stimulation was activated 1 month after implantation to separate out possible surgery‐associated effects from stimulation effects.^1^ Neuropsychological assessment was planned at baseline before implantation and 8, 16, 24, and 36 months after implantation. Outcome variables presented here refer to evaluation at short‐term (8 months) and last available follow‐up under stimulation in comparison to baseline assessment.

### 2.4 Seizure outcome

Seizure frequency in the month preceding the neuropsychological assessment as well as ASM were compared between baseline, short‐term and last follow‐up (percentage change of mean monthly seizure frequency).

## 3 RESULTS

Four patients underwent left temporal, three central (*N* = 2 right, *N* = 1 left), two left frontal, one left temporoparietal, and one left frontoparietal eFCS (all implantation sites were suspected to be eloquent cortex regions; therefore, no resective surgery was offered). At baseline assessment, six patients revealed impaired performance in lexical verbal fluency and eight in auditory working memory, which remained widely unchanged (see Table 2). At first follow‐up, four patients significantly worsened (RCI) in one cognitive parameter, which was not observed at long‐term follow‐up assessment anymore. The majority of assessed parameters remained overall stable or improved in some cases (for an overview see Table 2).

**TABLE 2:** Cognitive outcome at first and last available follow‐up.

Temporal left									Cognitive outcome							
Patient	Stimulation site	Language dominance	Age at epilepsy onset	Etiology	Age at surgery	Follow‐up interval after implantation	Percentage change of mean monthly seizure frequency	ASM change at FU	EpiTrack® score	Visuomotor speed	Cognitive flexibility	Auditory working memory	Verbal learning	Verbal delayed recall	Lexical verbal fluency	Naming
1	Temporal left	Left	9	FCD	23	7	−41.7%	Unchanged	Improved	Improved	Improved	Improved	Unchanged	Unchanged	Unchanged	Improved
2	Temporal left	Left	7	FCD	27	8	+560%	Unchanged	Unchanged	Unchanged	Unchanged	Unchanged	Improved	Unchanged	Unchanged	Improved
3	Temporal left	Left	22	Haemangiomatosis & malformation of cortical development	26	7 22	−33.3% −66.7%	LTG↓, PER+ OXC↓, BRV↑, PER↓	Unchanged Improved	Unchanged Unchanged	Deteriorated Unchanged	Improved Unchanged	(Not assessed) Unchanged	(Not assessed) Unchanged	Unchanged Unchanged	(Not assessed) Unchanged
4	Temporal left	Left	4	Remaining HS	24	8 35	−93.8% −34.9%	Unchanged BRV ↑	Unchanged Unchanged	Improved Improved	Improved Improved	Unchanged Unchanged	Improved Unchanged	Improved Unchanged	Deteriorated Unchanged	Unchanged Unchanged

Central region									Cognitive outcome										
Patient	Stimulation site	Language dominance	Age at epilepsy onset	Etiology	Age at surgery	Follow‐up Interval	Percentage change of mean monthly seizure frequency	ASM change at last Follow‐up	EpiTrack® Score	Visuomotor speed	Cognitive flexibility	Auditory working memory	Verbal learning	Verbal delayed recall	Lexical verbal fluency	Fine motor speed left hand	Fine motor speed right hand	Fine motor speed both hands	Fine motor speed both hands assembly
5	Central right	Unknown	2	FCD	35	8 35	−14.1% 21.80%	Unchanged CBZ↑	Unchanged Unchanged	Unchanged Unchanged	Unchanged Unchanged	Unchanged Improved	Unchanged Unchanged	Unchanged Unchanged	Unchanged Unchanged	Unchanged Unchanged	Unchanged Unchanged	Unchanged Deteriorated	Unchanged Unchanged
6	Central right	Unknown	6	FCD	44	7 15	−35.2% −57.6%	Unchanged Unchanged	Unchanged Unchanged	Unchanged Unchanged	Improved Improved	Improved Improved	Unchanged Unchanged	Improved Unchanged	Unchanged Unchanged	Unchanged Unchanged	Unchanged Unchanged	Unchanged Unchanged	Unchanged Unchanged
7	Central left	Unknown	0	No identified lesion	23	7 23	27.30% −9.1%	Unchanged Unchanged	Unchanged Unchanged	Deteriorated Unchanged	Unchanged Unchanged	Unchanged Unchanged	Unchanged Unchanged	Unchanged Unchanged	Unchanged Improved	(Not assessed) (Not assessed)	(Not assessed) (Not assessed)	(Not assessed) (Not assessed)	(Not assessed) (Not assessed)

Frontal left									Cognitive outcome										
Patient	Stimulation site	Language dominance	Age at epilepsy onset	Etiology	Age at surgery	Follow‐up interval	Percentage change of mean monthly seizure frequency	ASM change at last Follow‐up	EpiTrack® Score	Visuomotor speed	Cognitive flexibility	Auditory working memory	Verbal learning	Verbal delayed recall	Lexical verbal fluency	Semantic verbal fluency	Figural fluency (flexibility)	Interference	Visual planning
8	Frontal left	Left	0	FCD	27	7 35	−44.2% −29.9%	CLB↓, VPA+ LTG↑, CLB stopped	Unchanged Unchanged	Unchanged Unchanged	Unchanged Unchanged	Improved Unchanged	Unchanged Unchanged	Unchanged Improved	Unchanged Unchanged	Unchanged Unchanged	Unchanged Deteriorated	Improved Unchanged	Unchanged Unchanged
9	Frontal left	Bilateral	21	Heterotopia	25	8 36	−90% −90% (only 33 months available)	LCM↓ LCM↓, PER↑	Unchanged Unchanged	Unchanged Unchanged	Unchanged Unchanged	Unchanged Unchanged	Unchanged Unchanged	Unchanged Improved	Unchanged Unchanged	Unchanged Improved	Unchanged Unchanged	Unchanged Unchanged	Unchanged Unchanged

Other regions									Cognitive outcome										
Patient	Stimulation site	Language dominance	Age at epilepsy onset	Etiology	Age at surgery	Follow‐up interval	Percentage change of mean monthly seizure frequency	ASM change at last Follow‐up	EpiTrack® Score	Visuomotor speed	Cognitive flexibility	Auditory working memory	Verbal learning	Verbal delayed recall	Lexical verbal fluency	Semantic verbal fluency	Figural fluency (flexibility)	Visual working memory	Visuoconstruction
10	Temporo‐parietal left	Right	9	No identified lesion	28	7 36	−50% −50%	ZNS ↑ ESL↓	Improved Improved	Unchanged Unchanged	Improved Improved	Improved Improved	Unchanged Unchanged	Deteriorated Unchanged	Unchanged Unchanged	Unchanged Unchanged	Improved Improved	Unchanged Unchanged	Unchanged Improved
11	Fronto‐parietal right	Unknown	16	FCD	34	7 35	−63.6% −94.6%	LCM↓ LCM stopped	Improved Unchanged	Unchanged Deteriorated	Unchanged Unchanged	Unchanged Unchanged	Unchanged Unchanged	Unchanged Deteriorated	Unchanged Unchanged	Unchanged Unchanged	Unchanged Unchanged	Unchanged Unchanged	(Not assessed) (Not assessed)
									* *p* = 0.09 KW = 0.22	* *p* = 0.42 KW = 0.08	* *p* = 0.73 KW = 0.03	* *p* = 0.03 KW = 0.34 ^#^ *p* = 0.054 (T0–T1) ^a^ ^#^ *p* = 0.054 (T0–T2) ^a^ ^#^ *p* = 0.45 (T1–T2)	* *p* = 0.45 KW = 0.08	* *p* = 0.04 KW = 0.32 ^#^ *p* = 0.22 (T0–T1) ^#^ *p* = 0.47 (T0–T2) ^#^ *p* = 0.60 (T1–T2)	* *p* = 0.89 KW = 0.01				

Abbreviation: ASM, antiseizure medication; BRV, brivaracetam; CBZ, carbamazepine; CLB, clobazam; CNB, cenobamate; ESL, eslicarbazepine acetate; FCD, focal cortical dysplasia; HS, hippocampal sclerosis; LCM, lacosamide; LEV, levetiracetam; LTG, lamotrigine; OXC; oxcarbazepine; PER, perampanel; PHT, phenytoin; STM, short‐term memory; VPA, valproic acid, WM, working memory; ZNS, zonisamide. Cognitive outcome: highlighted results (improved/deteriorated) indicate reliable change (RCI ≥1.96 or ≤ −1.96) at 95% confidence.

^a^ Raw score increased.

* Friedman‐Test for test variables available for all patients, KW, Kendall's W.

^#^ If Friedman‐Test was significant, Wilcoxon‐Tests for post‐hoc comparisons were applied: *p*‐value (Holm corrected for multiple comparisons), T0, baseline, T1, first follow‐up, T2, last follow‐up.

The Friedman‐tests revealed a significant difference across the three time points for auditory working memory and verbal delayed recall (see Table 2). Post‐hoc Wilcoxon signed‐rank tests for auditory working memory performance just missed significance for the comparisons between baseline and first as well as last follow‐up after Holm correction (corrected *p* = 0.054 each, raw scores increased from baseline to follow‐up). Post‐hoc comparisons of verbal delayed memory performance were far from significance after correction for multiple comparisons (*p* > 0.2, see Table 2). All other comparisons did not show significant overall effects (see Table 2).

A closer look at individual performances depending on stimulation site revealed that patients receiving left temporal eFCS showed either stable or improved performance in the EpiTrack® (*N* = 2), visuomotor speed (*N* = 2), cognitive flexibility (*N* = 2), auditory working memory (*N* = 2), verbal learning (*N* = 2), verbal delayed recall (*N* = 1), and naming (*N* = 2). Two patients (#3 and #4, see Table 2) deteriorated in cognitive flexibility and lexical verbal fluency at first follow‐up, which was no longer seen at last assessment.

After central eFCS, one patient's bimanual fine motor speed and another patient's visuomotor speed deteriorated (#5 and #7, see Table 2). In both cases, these changes were only seen at one follow‐up assessment. Other functions were unchanged or, in some cases, improved at last follow‐up under stimulation (see Table 2).

After left frontal stimulation, one patient's (#8) figural fluency performance deteriorated at last assessment (first follow‐up unchanged). Both patients improved at last follow‐up in verbal delayed recall (see Table 2).

A patient with right‐sided frontoparietal eFCS (#11) showed a decline in visual motor speed as well as verbal delayed recall performance at last follow‐up (otherwise stable, EpiTrack Score improved at first follow‐up). One patient with left temporoparietal eFCS (#10) reported memory problems and revealed a transient decline in verbal delayed recall at first, but not at long‐term assessment and was otherwise stable (see Table 2). Deterioration at first follow‐up was seen under an increase of zonisamide (200 → 300 mg) and disappeared after it was reduced again.

Cognitive changes did not correspond to seizure outcome (p > 0.1). Out of seven patients who demonstrated decreased performance in one or more parameters, three had a reduction in seizure frequency of more than 50% (see Table 2). In one patient (#2) with a major transient increase in seizure frequency, cognitive performance was unchanged or even improved.

ASM remained stable between baseline and last available follow‐up in 4/11 patients. The medication of the remaining patients was optimized due to ongoing seizures (for details see Table 2). At group level, neither significant changes in the number of ASM (*p* > 0.2) nor defined daily dose (*p* > 0.7) were present.

## 4 DISCUSSION

Most investigated cognitive parameters remained unchanged after 7 months as well as almost 3 years of eFCS. Especially when stimulation occurred in regions where epilepsy surgery is typically associated with an elevated risk of deterioration, no systematic decline was observed. Whereas after resection of the left language‐dominant temporal lobe, impairments of verbal memory, naming, and verbal fluency are common,^6^ temporal eFCS did neither result in memory nor naming deficits, and only in one patient in a transient deterioration of verbal fluency.

In each patient, significant improvements in at least one parameter were detected at one follow‐up assessment. As most of the tests do not provide information on reliability for multiple follow‐up assessments, it remains unclear to what extent the improvements at the last follow‐up assessment may have been due to learning effects.

Other stimulation studies revealed similar results. Prior analysis of EpiTrack data available for 27 patients participating in the clinical trials did not show any deterioration.^2^ In a double‐blind, sham‐controlled, randomized, parallel‐group study of 5 days of fixed‐dose transcranial direct current stimulation (TDCS), 37 patients with well‐controlled TLE showed short‐term improvements in their depressive symptoms and no changes in memory performance 2 and 4 weeks later.^7^ A sham‐controlled, double‐blind, randomized study also found no cognitive changes (Mini‐Mental State Examination) 4 weeks after cathodal TDCS in 22 participants with focal epilepsy.^8^ Ten patients with intractable FLE even improved significantly in attention and working memory functions immediately and 1 month after 10 days of cathodal TDCS over their epileptogenic zone.^9^ Most of these studies have shorter follow‐up intervals as compared to our assessment with a median of 35 months.

Cognitive assessments with long follow‐up intervals have been reported for invasive stimulation methods. In a large double‐blind, randomized, sham‐controlled study,^10^ responsive neurostimulation above the seizure focus (mesial temporal or neocortical frontal or temporal) was not associated with cognitive decline after 2 years. The authors also found stable or even improved performance in naming (23.5%), verbal learning (6.9%), and memory performance (6.8%). Only for 1.4% (verbal learning), 0.7% (verbal delayed recall), and 6.7% (naming) of the patients was a significant decline (RCI 90%) reported. A review^11^ about the effects of invasive and noninvasive stimulation summarizes that cognition and mood remained largely stable under vagus nerve stimulation, responsive neurostimulation, trigeminal nerve stimulation, repetitive transcranial magnetic stimulation, and transcranial direct current stimulation, whereas after deep brain stimulation of the anterior thalamic nuclei some patients reported worsening of memory performance and depressive symptoms.^12^ However, standardized memory assessment did not show differences in reliable memory change between the active and control group at the end of the blinded phase after 4 months.^13^

In our patient group, 6/11 patients had a mean monthly seizure decrease of at least 50% at one or both (*N* = 3) follow‐up assessments (see Table 2). Increase of ASM was documented in three of these patients with an improved seizure situation at one time point (see Table 2). Therefore, it remains unclear to what extent eFCS independent of optimization of ASM helped to reduce seizures in these patients. Even one patient (#2) with an electrode defect and a major transient increase in seizure frequency up to +560% did not reveal significant cognitive worsening. Altogether, there was no systematic association found between changes in cognitive performance and seizure frequency (see Table 2). In none of the patients (#1 and 2) did discontinuation of treatment occur due to cognitive side effects.

ASM changes were made in five patients until first follow‐up assessment. A closer look revealed that in two of these patients one ASM (lacosamide, LCM) was reduced in dosage and the majority of the assessed parameters remained unchanged. In one of these patients, LCM could be completely tapered off under 95% of seizure reduction. Two of three patients with ASM increases showed deterioration in one cognitive parameter each: One patient reported memory problems under a higher dose of zonisamide (300 mg) and deteriorated in verbal delayed recall at first follow‐up (#10). It is well described that zonisamide can have negative effects on cognitive functions including verbal delayed recall.^14^ In the course of the study, verbal delayed free recall performance improved again after zonisamide was reduced back to 200 mg. The other patient (#3) was newly introduced to perampanel add‐on to a lower dose of lamotrigine and decreased in cognitive flexibility performance at that time. Usually, perampanel is not associated with cognitive side effects,^15^ only one study^16^ reported worsening of continuity of attention and speed of memory. However, it is well shown that every additional ASM is associated with more frequent cognitive side effects.^17^ The third patient (#8) had valproic acid newly introduced and simultaneously clobazam reduced at first follow‐up. Neither significant changes nor improvements of cognitive functions were observed.

There are some limitations: The present study is observational in nature and evaluated a limited number of patients undergoing unblinded, not sham‐controlled assessment with individually variable stimulation sites. Furthermore, ASM was not stable particularly with long‐term follow‐up. Cognitive assessment is thus needed in a larger patient cohort to corroborate these preliminary findings.

## 5 CONCLUSION

In this clinical observational study, no systematic cognitive changes were found in specific cognitive domains of the stimulated areas. The majority of patients showed neither significant cognitive changes after short nor longer follow‐up assessments under eFCS.

## CONFLICT OF INTEREST STATEMENT

KW has no conflict of interest to disclose. IG has no conflict of interest to disclose. JK has no conflict of interest to disclose. JL has no conflict of interest to disclose. BM has no conflict of interest to disclose. JB has no conflict of interest to disclose. BEAS serves as a scientific advisor for Precisis (Heidelberg, Germany) and has received a research grant from Ceregate (Hamburg, Germany) and travel support from Boston Scientific (Marlborough, MA, USA). VAC receives a collaborative grant from BrainLab (Munich, Germany). He serves as an advisor for Ceregate (Munich, Germany), Cortec (Freiburg, Germany), and Inbrain (Barcelona, Spain). He has an ongoing IIT with Boston Scientific (USA). He has received travel support and honoraria for lectures from Boston Scientific (USA). EK received speaker honoraria and financial compensation for travel expenses from Medtronic, UCB, Livanova, and Eisai and has participated in clinical trials for Medtronic, UCB, and Precisis. Her research is supported by the Deutsche Forschungsgemeinschaft (DFG KA 5786/2–1) as well as the Medical & Clinician Scientist Program (MCSP). SK has received speakers' honoraria from Bial, Eisai, Desitin, MerckSerono, UCB, and Zogenix. ASB received research support from Bial, Precisis, and UNEEG, and personal honoraria for lectures or advice from Angelini Pharma, Eisai, Jazz Pharmaceuticals, Precisis, UCB, and UNEEG. We confirm that we have read the Journal's position on issues involved in ethical publication and affirm that this report is consistent with those guidelines.

## Data Availability

The data that support the findings of this study are available on request from the corresponding author. The data are not publicly available due to privacy or ethical restrictions.
